# High prevalence of hepatitis B virus dual infection with genotypes A and G in HIV-1 infected men in Amsterdam, the Netherlands, during 2000-2011

**DOI:** 10.1186/1471-2334-13-540

**Published:** 2013-11-14

**Authors:** Antoinette C van der Kuyl, Fokla Zorgdrager, Boris Hogema, Margreet Bakker, Suzanne Jurriaans, Nicole KT Back, Ben Berkhout, Hans L Zaaijer, Marion Cornelissen

**Affiliations:** 1 Laboratory of Experimental Virology, Department of Medical Microbiology, Center for Infection and Immunity Amsterdam (CINIMA), Academic Medical Center of the University of Amsterdam, Meibergdreef 15, Amsterdam 1105, AZ, Netherlands; 2 Department of Blood-borne infections, Sanguin, Amsterdam, Netherlands; 3 Laboratory of Clinical Virology, Department of Medical Microbiology, Center for Infection and Immunity Amsterdam (CINIMA), Academic Medical Center of the University of Amsterdam, Meibergdreef 15, Amsterdam 1105AZ, Netherlands

## Abstract

**Background:**

Hepatitis B virus (HBV) is divided into 8 definite (A-H) and 2 putative (I, J) genotypes that show a geographical distribution. HBV genotype G, however, is an aberrant genotype of unknown origin that demonstrates severe replication deficiencies and very little genetic variation. It is often found in co-infections with another HBV genotype and infection has been associated with certain risk groups such as intravenous drug users and men having sex with men (MSM). We aimed to estimate the prevalence of HBV-G in the Netherlands by analysing samples from HBV-positive patients visiting the Academic Medical Center in Amsterdam.

**Methods:**

Ninety-six HBV-infected patients, genotyped as HBV-A or HBV-G infected, were retrieved from the clinical database. Blood plasma samples were analysed with a newly-developed real-time PCR assay that detects HBV-A and HBV-G. For three patients, the HBV plasma viral load (pVL) of both genotypes was followed longitudinally. In addition, three complete genomes of HBV-G were sequenced to determine their relationship to global HBV-G strains.

**Results:**

Ten HBV-G infections were found in the selected Dutch patients. All concerned HIV-1 infected males with HBV-A co-infection. Dutch HBV-G strains were phylogenetically closely related to reference HBV-G strains.

**Conclusions:**

In this study, HBV-G infection in the Netherlands is found exclusively in HIV-1 infected men as co-infection with HBV-A. A considerable percentage (37%) of men infected with HBV and HIV-1 are actually co- infected with two HBV genotypes.

## Background

Hepatitis B virus (HBV), the prototype member of the *Hepadnaviridae*, can cause chronic hepatitis, cirrhosis and ultimately hepatocellular carcinoma. HBV is currently divided into eight confirmed genotypes that differ >8% from each other at the nucleotide level: A to H (for a review, see: [1]). In addition, a novel genotype I has been reported from South-East Asia [2-5] and a candidate tenth genotype J was suggested [6]. Most genotypes can be split into subgenotypes that differ >4% from each other, such as HBV-A1 and HBV-A2 [7]. HBV-A2 is the most prevalent HBV variant in Western Europe and North America [7].

HBV genotype G (HBV-G), originally described as a mutated HBV variant that emerges during chronic HBV infection [8,9], was recognized as a separate genotype in 2000 [10]. HBV-G is distinct from other HBV genotypes in having a 36-nucleotide insertion at the 5′ end of the core protein gene, which adds 12 amino acids to the protein and interferes with core protein expression and virion secretion [11-13]. In addition, one or two stopcodons are usually present in the precore region that preclude HBeAg expression [10,14]. Altogether, these characteristics hamper virus replication and make HBV-G mono-infection a rarely observed event [14-16]. Apparently, HBV-G replication can be rescued by co-infection with another HBV genotype, and such dual infections have been observed repeatedly with HBV genotype A in Japan [17,18], the USA [19,20], Spain [21] and Canada [22], with HBV genotypes A, C or D in Germany [23,24], with HBV genotype H in Mexico [25], and with HBV genotype F in Argentina [26]. In fact, rescue by another genotype can be very effective, even to the point where HBV-G outcompetes the co-infecting strain [9,17,19]. This phenomenon has also been observed in humanized mouse models [27,28]. Infections with HBV-G are especially prevalent in specific risk groups such as human immunodeficiency virus type 1 (HIV-1) infected men having sex with men (MSM) and intravenous drug users [18,21,22,25,26,29-31]. HBV-G strains isolated worldwide show very little genetic variation compared to other HBV genotypes [10]. There are indications that mixed HBV infections that include an HBV-G strain are associated with increased liver fibrosis [27,28,30], suggesting that patients infected with HBV-G should be monitored more closely. Also, response to antiviral treatment has been reported to be less effective in patients co-infected with HBV-G as compared to single genotype infections [23].

The prevalence of HBV-G infections in most countries in northern Europe, including the Netherlands, is not known. Therefore, we determined the occurrence of HBV-G infections and its association with HBV-A in patients visiting our academic hospital in Amsterdam, the Netherlands during 2000-2011. HBV-A is the most prevalent, endemic HBV genotype in the Netherlands and is found especially among MSM [32]. In addition, we determined the longitudinal plasma viral load (pVL) changes of HBV in genotypes A and G in three dually infected patients. The complete genomes of three HBV genotype G strains were sequenced to analyse their genetic relatedness to genotype G strains isolated in other countries worldwide.

## Methods

### Patient materials

A total of 1674 patients tested HBsAg positive in the AMC (Amsterdam, the Netherlands) during 2000-2011, of which 1008 were also tested for HIV-1 infection (HBV tests requested from outside the AMC, but performed here were not included in the numbers). Of those 1008 HBV positive patients, 211 were found to be also HIV-positive. Patients receiving anti-HBV treatment in whom therapy failure was observed and drug resistance was thus suspected (N = 343), HBV genomic fragments were amplified, sequenced, genotyped and analysed for drug-resistance mutations. For this study, we selected AMC patients from the database of the AMC Laboratory of Clinical Virology (Department of Medical Microbiology) that previously were found to be infected with HBV genotype A or G. HBV genotyping was done with an in-house sequencing assay. The Laboratory of Clinical Virology participates in the Quality Control for Molecular Diagnostics Hepatitis B virus Genotype EQA Programme [33] to ensure adequate performance of this genotyping assay. A total of 96 patient entries met these criteria and had sample availability; 91 had been diagnosed as HBV genotype A and 5 as HBV genotype G. Twenty-seven were HIV-positive (all males), 54 were HIV-negative and 15 had an unknown HIV status. In total, sixty-nine patients were male, and 27 were female. Of the 69 included men, 25 originated from the Netherlands; the others were from Suriname (N = 11), Africa (N = 8), other European countries (N = 5), or of unknown or other origin (N = 20). Of the 27 female patients, 6 were from the Netherlands, 15 from Suriname, 4 from Africa, and for 2 the country of origin was unknown.

### Real-time PCR assay

Viral DNA was isolated from blood plasma samples with the QIAamp UltraSens Virus Kit (QIAGEN, the Netherlands). Single-tube real-time PCR reactions that specifically quantify HBV genotype A or G DNA were developed in the HBV X-protein gene and analysed on an ABI PRISM® 7000 Sequence Detection System (Applied Biosystems, Foster City, CA, USA) using the primers and probes shown in Table 1. Primers and probes were highly specific for the targeted HBV genotype (either A or G). Cross-reactivity was not detected as the primers and probes were able to amplify 5 copies of the targeted genotype in a background of up to 10^8^ copies of the non-targeted genotype. All subgenotypes of HBV-A can be quantified with this assay. The lower limit of detection is 5 copies per reaction (= 1000 copies/ml).

**Table 1:** Primers and probes used in the HBV-A and HBV-G genotyping assay

**Primer**	Position in HBV genome*	**Sequence (5′ → 3′)**
5*'*HBV-A-TM3_2	1585-1606	TTC GCT TCA CCT CTG CAC GTT G
3*'*HBV-A-TM3	1669-1647	CCA AGA GTC CTC TTA TGT AAG AC
3*'*HBV-A-probe-TM3_2	1636-1613	ATC TGA TGG GCG TTC ACG GTG GTC
5*'*HBV-G-TM3_2	1585-1606	TTC GCT TCA CCT CTG CAC GTT A
3*'*HBV-G-TM3	1669-1647	CCA AGA GTC CTC TTA TAT AAC TG
3*'*HBV-G-probe-TM3_2	1636-1613	ATG ATG AGA GGT GTT CAT GGC GGT T

*Numbering starting at the EcoR1 site.

### HBV-G genome sequencing

HBV genotype G genome fragments were amplified from viral DNA isolated from plasma samples and directly sequenced with the BigDye Terminator cycle sequencing kit (Applied Biosystems, Foster City, CA, USA). Electrophoresis and data collection were performed on an ABI PRISM 3100 genetic analyser (also from Applied Biosystems). Sequences were assembled with CodonCode Aligner [34], and aligned with reference HBV sequences from the NCBI nucleotide database [35] using ClustalW implemented in BioEdit Sequence Alignment Editor version 7.0.9 [36]. GenBank accession numbers for the full-length genotype G sequences are: KF767450, KF767451, and KF767452.

### Ethics

No specific approval by the Institutional Review Board (IRB) of the Academic Medical Center (AMC) of the University of Amsterdam was needed for this study in the Netherlands, because the body materials for this study were collected under medical treatment, and can be used for further scientific research when the patients have given consent and samples are analysed anonymously, as was the case here (Code of Conduct as implemented by the Committee on Regulation of Research COREON of the Netherlands Epidemiological Society and the Dutch Federation of Biomedical Scientific Societies that is followed by the IRB of the AMC).

## Results

### Prevalence of HBV-A and -G in Amsterdam

**Figure 1:**
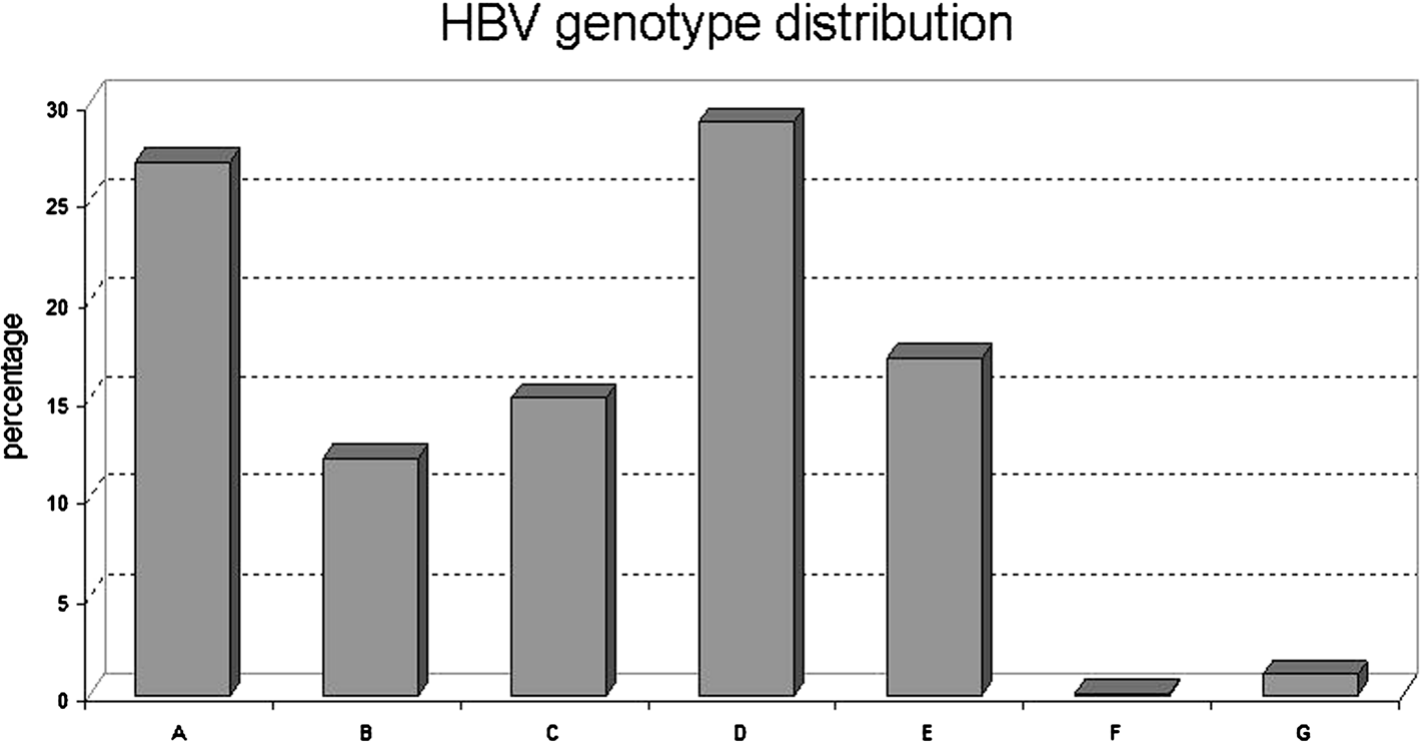
**Percentages of HBV genotypes A-G in HBV-infected patients from Amsterdam.** HBV genotypes were determined by the Laboratory of Clinical Virology of the AMC (Amsterdam, the Netherlands) during 2000-2011.

The prevalence of HBV genotypes as determined by the Laboratory of Clinical Virology in HBV-infected patients visiting the AMC is shown in Figure 1. Genotypes D (29%) and A (27%) were the most prevalent, followed by genotypes E (16%), C (15%) and B (12%). Genotype F or G infections are uncommon (≤ 1%).

A total of 96 patients with HBV infection genotype A or G infection for whom samples were available were assessed for the presence of single (A or G) or A/G dual HBV infection with a single-tube real-time PCR assay that specifically quantifies these genotypes. Patient characteristics and results are summarized in Table 2. All female patients (N = 27) were found to be singly infected with HBV-A. Of the 69 male patients, 55 were infected with HBV-A only, but 10 were dually infected with both HBV-A and -G. According to the clinical database all 10 dual infected patients were infected with the HBV subgenotype A2. In four male patients, the HBV pVL was below the detection limit of the assay. None of the patients exhibited a single infection with HBV-G; all 5 patients previously diagnosed as HBV genotype G also carried genotype A. Five patients previously diagnosed as genotype A also carried genotype G. Strikingly, all ten dually HBV-A/G infected patients were also infected with HIV-1. In contrast, none of the HIV-1 negative patients (42 males and 27 females) were dually HBV infected. So, HBV-A/G dual infection is significantly associated with HIV-1 infection (two-tailed Fisher’s exact test, p < 0.0001). In total, 37% of HBV/HIV-1 infected patients harboured two HBV genotypes. All ten dually HBV infected patients were HBeAg positive at the time of testing with the AxSYM® HBe Assay (Abbott Diagnostics, Lake Forest, Ill, USA), suggestive of infection with a precore protein producing strain [19], such as HBV-A [21]. The majority of HIV-1 infected males were of Dutch ethnicity (13/23), with an equal distribution between those singly (7/13) or dually HBV (6/10) infected. For the other HIV-1 infected patients the origin was either unknown (4/23) or varied widely (6/23, from the Dominican Republic, Ecuador, Dutch Antilles, Ethiopia, and Germany). Of the HBV-A/G dually infected patients, five had unknown risk factors for infection, 1 reported heterosexual contacts as a risk factor, and four were labelled as MSM in the database.

**Table 2:** Patient characteristics and results

**Gender**	**N**	**HIV-1 status**		**HBV real-time PCR**	
Male	69	Negative:	N = 32	HBV-A:	N = 55
		Positive:	N = 27	HBV-A + HBV-G:	N = 10
		Unknown:	N = 10	Undetectable:	N = 4
Female	27	Negative:	N = 22	HBV-A:	N = 27
		Positive:	N = 0	HBV-A + HBV-G:	N = 0
		Unknown:	N = 5	Undetectable:	N = 0
Total	96	Negative:	N = 54	HBV-A:	N = 82
		Positive:	N = 27	HBV-A + HBV-G:	N = 10
		Unknown:	N = 15	Undetectable:	N = 4

Thus, HBV-G infection in Dutch patients is strongly associated with HBV-A and HIV-1 co-infection, which likely explains the male-specificity in this analysis, as the majority of HIV-1 infected patients in the Netherlands are male (80% in 2011), of which 73% belong to the MSM risk group [37]. These results suggest that HBV-G is primarily circulating among MSM in the Netherlands.

To assess the possibility that both HBV-G and HIV-1 circulate in a certain subgroup of patients, as well as investigating a potential simultaneous transmission of both viruses we analysed HIV-1 polymerase sequences that were available for 6 out 10 HIV-1/HBV-A/G infected patients and 10 HIV-1/HBV-A2 infected patients that were not infected with HBV-G. A phylogenetic tree was generated of these 16 sequences plus reference sequences of HIV-1 subtypes A-K with the neighbour-joining option in MEGA5 based upon a Kimura 2-parameter distance matrix [38]. The NJ tree suggested no significant relationship between the HIV-1 polymerase sequences of the 6 HBV-A/G dual infected patients (not shown) suggesting that transmission of HIV-1 and HBV-G are independent events.

### Complete HBV-G genome analysis

To investigate whether HBV-G strains circulating in the Netherlands are similar to global HBV-G strains, the DNA genome of three virus isolates (from patients 7, 23 and 27, all of Dutch ethnicity) were amplified and completely sequenced. The sequences demonstrated the characteristic 36-nucleotide insertion at the 5′ end of the core protein gene and two stopcodons in the precore region that preclude HBeAg expression. Also, a fourth, but incompletely sequenced HBV-G genome from patient 26 (originating from the Dominican Republic, but infected in the Netherlands, where he presented with an acute HBV infection) showed high sequence similarity with the other Dutch strains (not shown). HBV-G strains of patients 7 and 27 were closely related to each other and to an HBV-G strain originating from a Dutch blood donor with a HBV-G mono-infection [14]. The virus isolates of both patients showed resistance mutations against 3TC in the RT region (L180M and M204V).

**Figure 2:**
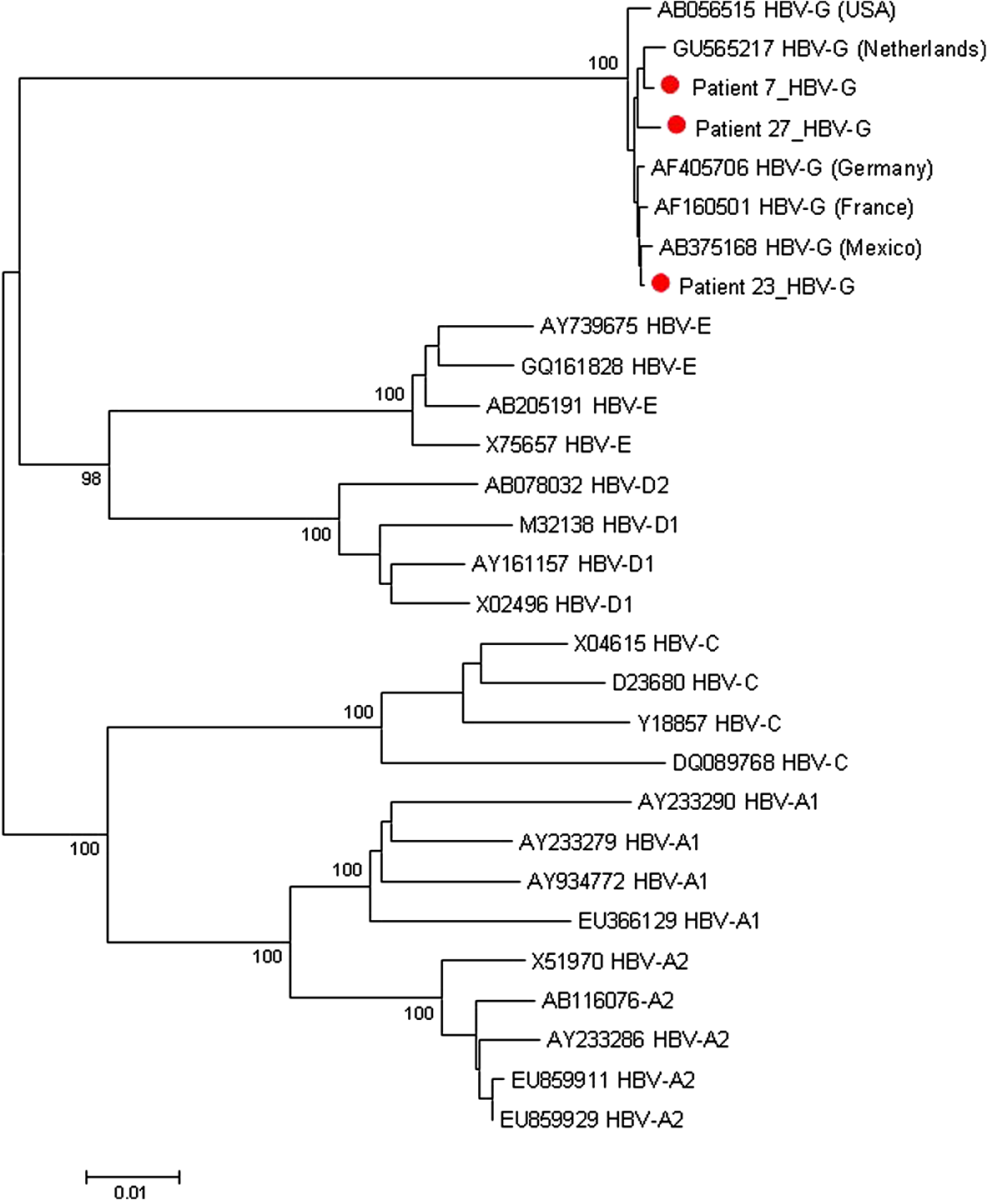
**Phylogenetic analysis of full-length HBV sequences.** The neighbour-joining tree was generated with the MEGA5 software package [38] and shows the relationship between three novel, full-length HBV-G strains from the Netherlands (indicated by red circles) and reference sequences of HBV genotypes A1, A2, C, D1, D2 and G obtained from the NCBI nucleotide database [35]. Distances were estimated with the Kimura 2-parameter method, and bootstrap resampling was done with 1000 replicates. The accession number of the reference sequences is indicated together with the HBV genotype.

All three completely sequenced Dutch HBV-G strains clustered with HBV-G reference sequences in a phylogenetic analysis (Figure 2). Genetic distances between the Dutch HBV-G genomes and reference HBV-G sequences were extremely low, and ranged between 0.002-0.008 for the full-length sequences. This is in line with the low genetic diversity of less than 0.5% detected for all HBV-G strains isolated worldwide [39].

For the other 7 patients, a 359 nucleotide fragment spanning the precore/core region of HBV-G was amplified and sequenced. All 7 isolates contained the typical 36-nucleotide insertion as well as the two stopcodons, and clustered with HBV-G sequences.

### HBV-A and -G plasma viral load

**Figure 3:**
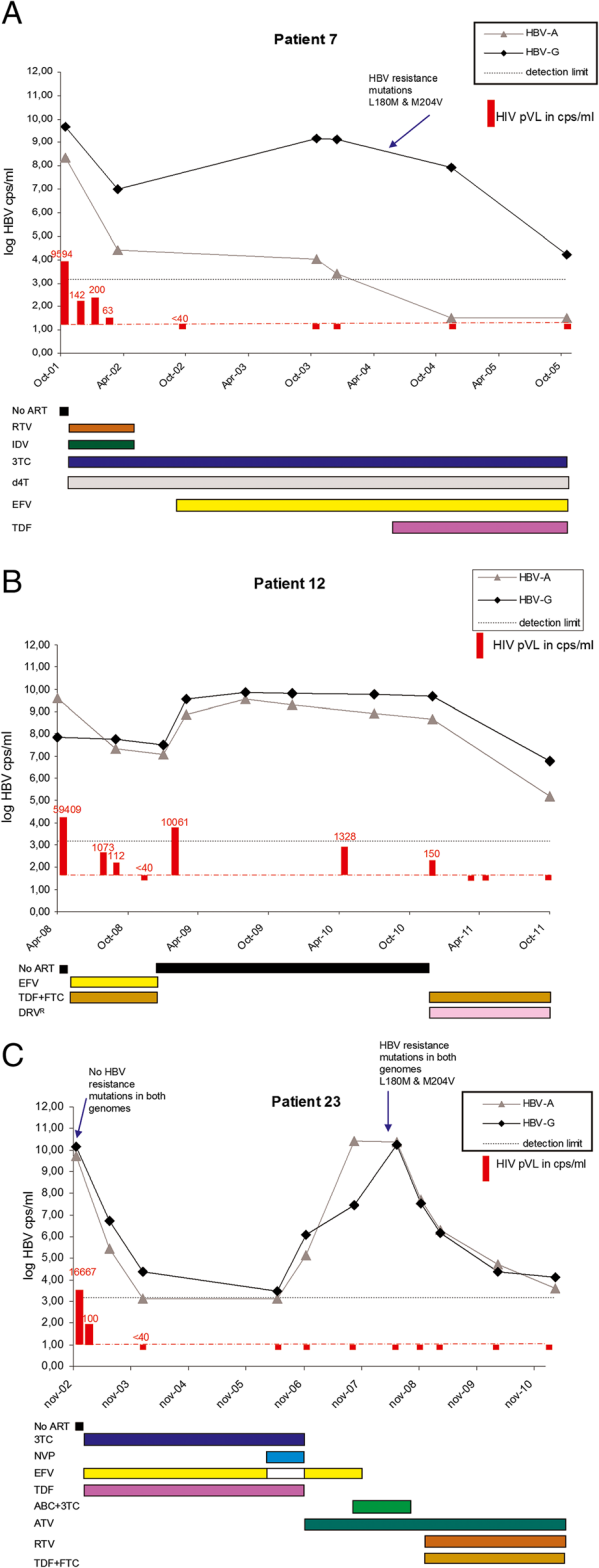
**Longitudinal analysis of the HBV-A and -G pVL in three male HIV-1 infected patients.** Panel **A** = patient 7; panel **B** = patient 12; panel **C** = patient 23. The HIV-1 pVL is indicated with red bars; numbers in red are HIV-1 copies/ml. ART regimens are shown below the graphs. Abbreviations: 3TC = lamivudine; ABC = abacavir; ATV = atazanavir; d4T = stavidine; DRV = darunavir (boosted with RTV); EFV = efavirenz; FTC = emtricitabine; IDV = indiniavir; NVP = nevirapine; RTV = ritonavir; TDF = tenofovir. 3TC and TDF are also effective against HBV.

For three HBV-A/G dually infected patients (nos. 7, 12 and 23), serial blood plasma samples were available for a longitudinal analysis. In all three patients, both genotypes were already present in the first available sample, so that the order of infection could not be established. A longitudinal analysis of the pVL of each HBV genotype was performed with the real-time PCR assay (Figure 3). All three patients were also HIV-1 infected, and treated with antiretroviral therapy, including lamivudine (3TC) and tenofovir (TDF) that target both the HIV-1 and the HBV RT enzymes. In general, the pVL of HBV-A and HBV-G follow a similar course in these patients, increasing or decreasing in parallel.

In patient 7, the HBV-G pVL exceeded the HBV-A pVL considerably (>10^5^ difference) at all time-points over a 4-year period (Figure 3A). The HBV-A and HIV-1 pVL decreased significantly after the start of an ART regimen containing 3TC in 2001, and remained close to or below the detection limit of the assay afterwards for HBV-A (Figure 3A). 3TC drug resistance occurred in the HBV-G strain, and its pVL rebounded after an initial drop. Subsequent initiation of TDF treatment (in addition to 3TC) resulted in a significant decrease in the HBV-G pVL.

In patient 12, the HBV-G pVL exceeded the HBV-A pVL at least tenfold over a 42 month period, except in the first sample measured (Figure 3B). A period without ART resulted in an increase in HBV pVL of both genotypes that decreased again with the restart of ART that included TDF (Figure 3B).

In patient 23, the pVL of the two genotypes was comparable over an 8-year period, despite dramatic fluctuations due to therapy changes (Figure 3C). The HBV-A and -G pVL also decreased sharply with the start of an ART regimen that contained both 3TC and TDF. However, mutations associated with 3TC resistance (L180M and M204V) developed three years later in both genotypes. A regimen containing TDF was subsequently installed, which lowered both HBV pVLs to just above the detection limit of the assay.

## Discussion

In a population of 96 HBV-infected patients visiting our academic hospital in Amsterdam that had been genotyped to harbour HBV-A or HBV-G, 10 HBV-A/G dual infections were detected. All 10 HBV-G infections concerned men that were also infected with HIV-1. Studies from other countries report a similar association of HBV-G infection with HIV-infection and with the MSM risk group [22,25,26,30,31]. In our study, only four HBV-A/G infected patients could be positively identified as MSM, for five others the risk factor was not stated. But as HBV-A is largely spreading in MSM (as is HIV-1) in the Netherlands; their HBV-G infection is most likely also due to homosexual contacts.

In contrast to the first report of HBV-G in the Netherlands that occurred as a mono-infection, the HBV-G infections detected in this study all represent co-infections with HBV-A. The initial mono-infection occurred in a Dutch male blood donor who denied risk factors for HBV infection, suggesting that HBV-G is also present outside the MSM risk group [14]. However, drug-resistance mutations were present in this HBV-G isolate, implying that the strain was most likely transmitted from an ART-treated individual.

HBV-G infections are mainly noticeable after infection with a “helper” HBV strain, here genotype A, and especially during HIV-1 co-infection that decreases HBV immune control and increases HBV replication [40]. HBV-G mono-infected patients have been reported to be HBeAg negative, variably HBsAg positive (2 out of 3 cases), while HBcAb and HBsAb levels can be inconsistent and/or delayed [14-16]. So, it is not always easy to recognize an HBV-G mono-infected patient as being HBV positive, especially in a routine diagnostic setting, and these patients might not have been included in the current study selection. It would be interesting to examine HIV-1 infected HBV-negative MSM for HBV-G mono-infection, as both viruses are circulating in this risk group. The newly-developed real-time PCR assay has a detection limit of 5 copies per reaction, enabling the detection of the low viral DNA levels as observed in HBV-G mono-infection.

Full-length genome sequencing indicated that the newly characterized Dutch HBV-G strains are closely related to reference HBV-G strains isolated around the world. They all contained the genome peculiarities associated with HBV-G, e.g. the 36-nucleotide insertion at the 5′ end of the core protein gene and two stopcodons in the precore region, as did isolates from 7 other patients. This again indicates that HBV-G has no specific geographical distribution as is the case for the other HBV genotypes.

For three patients, longitudinal blood plasma samples were available. Those samples were analysed with the real-time PCR assay to measure the relative contributions of each genotype to the pVL. In one patient (no. 23), HBV-A and HBV-G replicated at a similar level. In the other two patients (no. 7 and no. 12), the HBV-G pVL exceeded the HBV-A pVL at least 10 times, in line with other reports [9,17,19]. The increased HBV-G load in the presence of a helper strains is also in line with the results from humanized mouse models [27,28]. In all three cases, the patients were already infected with both HBV genotypes and HIV-1 at the first available sampling time, so that the order of infection cannot be determined. It is possible that the infections occurred simultaneously, as 37% of the Dutch HIV-1/HBV-infected MSM were found to be co-infected with HBV-A and HBV-G, and could thus transmit multiple HBV strains simultaneously. However, in the dual HBV-A/G infections described here, the HBV-G pVL exceeds the HBV-A pVL at least 10 times at most time points, so it is likely that HBV-G has a better chance to be transmitted. This finding of a much increased HBV-G pVL in A/G dual versus mono-infections could also explain why 5 patients were determined as being singly infected with HBV-G using the clinical HBV genotyping assay.

## Conclusions

The prevalence of HBV genotype G in the Netherlands was investigated using real-time PCR assays. HBV-G infection was found in 10/96 individuals previously identified as infected with HBV genotype A or G. These 10 patients were all HIV-1 infected men that were also infected with a HBV genotype A strain, more specifically the HBV-A2 genotype that is endemic in the Netherlands. These findings suggest a strong association of HBV-G in the Netherlands with the HIV-1 infected male risk group, as has been reported from other countries.

## Abbreviations

HBV: Hepatitis B virus; HIV-1: Human immunodeficiency virus type 1; MSM: Men having sex with men; pVL: Plasma viral load.

## Competing interests

The authors declare that they have no competing interests.

## Authors’ contributions

ACvdK drafted the manuscript, FZ performed the experiments, BH was involved in sequencing, MB performed the database selections with the help of SJ and NKTB, BB and HLZ critically revised the manuscript, and MC designed the experiments. All authors read and approved the final manuscript.

## Pre-publication history

The pre-publication history for this paper can be accessed here:

http://www.biomedcentral.com/1471-2334/13/540/prepub
